# Advancing Brain-Computer Interface Closed-Loop Systems for Neurorehabilitation: Systematic Review of AI and Machine Learning Innovations in Biomedical Engineering

**DOI:** 10.2196/72218

**Published:** 2025-11-05

**Authors:** Christopher Williams, Fahim Islam Anik, Md Mehedi Hasan, Juan Rodriguez-Cardenas, Anushka Chowdhury, Shirley Tian, Selena He, Nazmus Sakib

**Affiliations:** 1 Department of Computer Science, Troy University, Troy, AL, United States; 2 Department of Mechanical Engineering, Khulna University of Engineering and Technology, Khulna, Bangladesh; 3 College of Computing and Software Engineering, Kennesaw State University, Marietta, 1100 South Marietta Pkwy SE, Marietta, GA, 30067, United States, 1 470-578-3803; 4 Lambert High School, Suwanee, GA, United States

**Keywords:** Brain-Computer Interface, closed-loop systems, Artificial Intelligence, AI, Machine Learning, neurorehabilitation, cognitive monitoring, real-time monitoring, healthcare technology, biomedical signal processing, human-computer interaction, PRISMA

## Abstract

**Background:**

Brain-computer interface (BCI) closed-loop systems have emerged as a promising tool in health care and wellness monitoring, particularly in neurorehabilitation and cognitive assessment. With the increasing burden of neurological disorders, including Alzheimer disease and related dementias (AD/ADRD), there is a critical need for real-time, noninvasive monitoring technologies. BCIs enable direct communication between the brain and external devices, leveraging artificial intelligence (AI) and machine learning (ML) to interpret neural signals. However, challenges such as signal noise, data processing limitations, and privacy concerns hinder widespread implementation.

**Objective:**

The primary objective of this study is to investigate the role of ML and AI in enhancing BCI closed-loop systems for health care applications. Specifically, we aim to analyze the methods and parameters used in these systems, assess the effectiveness of different AI and ML techniques, identify key challenges in their development and implementation, and propose a framework for using BCIs in the longitudinal monitoring of AD/ADRD patients. By addressing these aspects, this study seeks to provide a comprehensive overview of the potential and limitations of AI-driven BCIs in neurological health care.

**Methods:**

A systematic literature review was conducted following PRISMA (Preferred Reporting Items for Systematic Reviews and Meta-Analyses) guidelines, focusing on studies published between 2019 and 2024. We sourced research articles from PubMed, IEEE, ACM, and Scopus using predefined keywords related to BCIs, AI, and AD/ADRD. A total of 220 papers were initially identified, with 18 meeting the final inclusion criteria. Data extraction followed a structured matrix approach, categorizing studies based on methods, ML algorithms, limitations, and proposed solutions. A comparative analysis was performed to synthesize key findings and trends in AI-enhanced BCI systems for neurorehabilitation and cognitive monitoring.

**Results:**

The review identified several ML techniques, including transfer learning (TL), support vector machines (SVMs), and convolutional neural networks (CNNs), that enhance BCI closed-loop performance. These methods improve signal classification, feature extraction, and real-time adaptability, enabling accurate monitoring of cognitive states. However, challenges such as long calibration sessions, computational costs, data security risks, and variability in neural signals were also highlighted. To address these issues, emerging solutions such as improved sensor technology, efficient calibration protocols, and advanced AI-driven decoding models are being explored. In addition, BCIs show potential for real-time alert systems that support caregivers in managing AD/ADRD patients.

**Conclusions:**

BCI closed-loop systems, when integrated with AI and ML, offer significant advancements in neurological health care, particularly in AD/ADRD monitoring and neurorehabilitation. Despite their potential, challenges related to data accuracy, security, and scalability must be addressed for widespread clinical adoption. Future research should focus on refining AI models, improving real-time data processing, and enhancing user accessibility. With continued advancements, AI-powered BCIs can revolutionize personalized health care by providing continuous, adaptive monitoring and intervention for patients with neurological disorders.

## Introduction

The adoption of technology in health care and wellness monitoring has grown significantly in recent years [1,2]. As of 2024, more than 1.3 billion people worldwide relied on digital health tools such as fitness trackers, smartwatches, and virtual doctor consultations. In the United States alone, 43% of the population actively used health apps [3,4]. This surge in digital health adoption is further reflected in the health care IT market, which is projected to expand from US$360 billion in 2024 to over US$730 billion by 2029 [5]. A recent survey revealed that 80% of Americans own at least one such device, including blood pressure monitors (45%), electric toothbrushes (39%), and fitness trackers or pedometers (24%) [6]. These devices play a crucial role in early detection and management of health conditions; notably, 28% of users reported receiving alerts about potential health issues from their devices, leading to successful diagnoses after consulting with health care professionals [6].

As technological advancements continue to reshape health care, their role in the early detection and management of Alzheimer disease and related dementias (AD/ADRD) is becoming increasingly critical [7]. AD is known as a neurological disorder characterized by memory loss, cognitive decline, and impaired motor skills [8]. It damages brain cells responsible for important mental functions and enables the cells themselves to degenerate and die. The degeneration begins from cognitive impairments, with motor functions still intact. Gradually, over time, this progresses into neuronal degeneration in several areas of the brain, including the hippocampus and mediotemporal cortex [9]. The disease is most commonly found in older adult populations; the prevalence of all dementias is known to increase for people aged 60‐90 years, making aging the biggest risk factor for AD [10]. While the disease is irreversible and has no cure, early detection and continuous monitoring can significantly improve patient outcomes. However, up to a third of dementia cases remain undiagnosed, and existing diagnostic methods are often slow and inaccurate [11]. The integration of technology—through wearable devices, advanced diagnostic tests, and AI-driven analysis—enables continuous monitoring and early identification of cognitive decline.

A promising innovation in this landscape is brain-computer interfaces (BCIs), which have the potential to revolutionize the diagnosis and management of neurodegenerative diseases like AD/ADRD [12,13]. BCIs have been the subject of significant research due to their correlation to decoding neural activity and use by people with disabilities. The BCI closed-loop system directly connects the human brain and the outside environment [14], allowing for direct communication between a person and a computer. It enables users the ability to operate external devices through their brain activity and translate brain signals, strictly produced by the central nervous system, into commands that carry out a desired action [15]. The “closed-loop” aspect allows for the use of real-time data to monitor and adjust updates based on the patient’s condition. In particular, BCI applications have been initially designed to help people with disabilities and enhance neuroplasticity, characterized as the capacity of the brain to change or adapt its morphology in response to experiences [16]. The system may also help in rehabilitation for people with strokes, head trauma, and other disorders [15]. Broadly, a BCI system consists of 4 standard, sequential components: signal acquisition, feature extraction, feature translation, and device output [15]. Within each component, there exist several methods and techniques that have been reviewed that effectively execute the goal of detecting and qualifying features of brain signals. There are many parameters that the BCI closed-loop system seeks to measure, with the intention of collecting large and diverse datasets; performance metrics heavily influence the quality of BCI research, which several methods of BCI closed-loop systems depend on.

BCIs facilitate direct communication between the brain and external devices, allowing real-time monitoring of neural activity and cognitive function. This technology is particularly valuable for detecting early neurophysiological changes that precede noticeable cognitive decline, offering a more objective and continuous assessment than traditional diagnostic methods [17,18]. By integrating BCIs with artificial intelligence (AI) and machine learning (ML), researchers can analyze brain signals to identify patterns associated with Alzheimer progression, potentially enabling earlier and more accurate diagnoses. Furthermore, BCIs hold promise for enhancing cognitive rehabilitation and assistive communication for patients in later stages of the disease. As the demand for advanced neurological monitoring grows, BCIs represent a critical step toward personalized and proactive dementia care, bridging the gap between early intervention and improved patient outcomes [19-21]. Therefore, in the context of neuroscience and AI, the BCI is a proposed solution for identifying and providing neurorehabilitation methods through decoding electroencephalogram (EEG) signals. This can prove to be of great significance for the detection and diagnosis of several neurological disorders, such as Alzheimer disease, through exploiting the use of neuron devices and stimulating biological sensory neurons [22]. The ultimate motivation is to integrate AI models and BCI systems in order to allow for personalized treatment plans and contribute greatly to breakthroughs in health care.

However, many limitations are associated with BCI-based closed-loop systems that can hinder the systems’ performance and efficacy. For instance, BCI applications must recalibrate the system in order to account for each user/participant due to the high variability in brain signals [23].

The model must be trained from scratch each time there is a new subject. This contributes to significant financial expenses. Furthermore, the limited size of datasets can lead to overfitting, which occurs when a model fits too closely to its training data rather than including new data [23]. When using an EEG to capture brain signals, several limitations exist with using the method. EEG-based BCI systems measure the average activity of neurons with electrodes located on the surface of the brain [23]. These generally produce a low signal-to-noise ratio (SNR); a low SNR indicates that the signal is corrupted by noise and therefore makes it difficult to interpret brain signals. This review analyzes several solutions to these challenges with the use of machine learning algorithms and networks that can easily decode complex brain data. However, this field of research is not limited to current knowledge and there is still more to explore regarding the use of machine learning and deep learning in BCI closed-loop systems.

**Figure 1.**
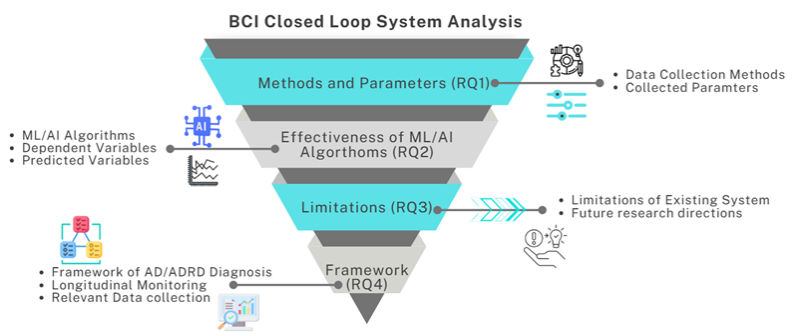
Brain-computer interface closed-loop systems overview in health care and wellness monitoring. AD/ADRD: Alzheimer disease/Alzheimer disease and related dementias; AI: artificial intelligence; BCI: brain-computer interface; ML: machine learning;

In the exploration of BCI systems and artificial intelligence algorithms, our research aims to address a range of critical questions and topics that are integral to advancing this field, as shown in Figure 1. By investigating the following research questions, we will gain a comprehensive understanding of the real-world applications of BCIs, uncovering insights that could lead to innovative opportunities and improvements in the monitoring of AD/ADRD patients.

RQ 1. What specific methods and parameters are used in the BCI closed-loop system?

RQ 2. How effective are the different ML and AI algorithms used in the BCI closed-loop system?

RQ 3. How can we critically investigate the limitations in the development and implementation of the BCI closed-loop system?

RQ 4. How can we design a BCI closed-loop system-based framework for longitudinal monitoring of AD/ADRD patients?

The remainder of the paper is organized into 5 key sections. Section 2 outlines the methodology, comprising three subsections that detail the scoping criteria, literature search strategy, and data analysis procedures. Sections 3, 4, 5, and 6 address the 4 research questions in depth. Finally, Section 7 concludes the literature review, summarizing the key findings and their implications.

## Methods

### Overview

Our research approach centered on a comprehensive evaluation of the literature exploring the integration of AI—particularly its subset, ML—within BCI closed-loop systems in health care. The goal was to synthesize current knowledge on the methodologies, algorithms, outcomes, limitations, and emerging directions that define this interdisciplinary field. To achieve this, we developed a targeted search strategy using relevant keywords and Boolean operators, enabling us to identify both theoretical advancements and real-world applications of AI- and ML-enhanced BCIs. This method allowed for a focused analysis of how these technologies are transforming neurological monitoring, cognitive rehabilitation, and personalized patient care.

### Scoping Criteria

Our scoping criteria focused on the specific domain of BCI closed-loop systems integrated with ML and AI in health care. We prioritized studies published between 2019‐2024 to ensure the relevance and timeliness of our findings. Our approach included not only technological advancements but also practical challenges and developments in BCI closed-loop systems in health care. Specifically, we reviewed studies that examined the various methods and parameters collected in BCI closed-loop systems (RQ1), ensuring a comprehensive understanding of data acquisition, preprocessing, and real-time feedback mechanisms. We also investigated the ML and AI algorithms used, and the outcomes obtained (RQ2), identifying the overall effectiveness of these algorithms in clinical and experimental settings. In addition, we focused on studies discussing the limitations encountered in current BCI closed-loop systems and proposed future research directions (RQ3), aiming to understand the barriers to implementation, ethical considerations, and technological limitations (RQ4). Exclusion variables from some papers were added, as through our search, we filtered out papers that were not relevant to our goals, any research conducted on animals and not humans, and a lack of focus on Machine Learning.

### Systematic Literature Search

Our systematic approach involved gathering, critical assessing, integrating, and presenting findings from various research papers on BCI closed-loop systems integrated with ML and AI in health care. We followed a detailed procedure to conduct and report systematic literature reviews, ensuring a rigorous selection process. Initially, we developed a carefully crafted search query to refine our search effectively, using terms such as “BCI OR brain computer interface,” “AND Machine Learning OR AI OR algorithm,” “AND Alzheimer OR Dementia.” Boolean operators like “AND” and “OR” were used strategically to narrow our search. This search spanned 4 major databases: PubMed, IEEE, ACM (Association for Computing Machinery), and Scopus. From these databases, we identified a total of 220 papers: 43 from PubMed, 22 from IEEE, 114 from ACM, and 41 from Scopus. After removing 8 duplicate records, 212 unique records were screened. During the screening phase, 179 records were excluded for reasons such as being out of context (n=84), not relevant to the research questions (n=94), or inaccessible (n=1). Subsequently, the titles and abstracts of the 212 screened records were assessed for eligibility, resulting in 33 full-text articles being reviewed. Of these, 15 reports were excluded due to being theses or books (n=9), report articles (n=4), or of poor quality (n=2). Ultimately, 18 studies met all inclusion criteria and were included in the final review. These papers were selected based on their focus on BCI closed-loop systems in health care, the integration of ML and AI, and their relevance to our study. We prioritized papers displaying rigorous methodologies, including empirical studies, surveys, case studies, experiments, and systematic literature reviews, showcasing innovative approaches, novel insights, or significant findings. In addition to the primary search, we cross-referenced each article’s citations to identify other pertinent papers, ultimately including any that fit our criteria.

## Results

### Study Selection and Characteristics

**Figure 2.**
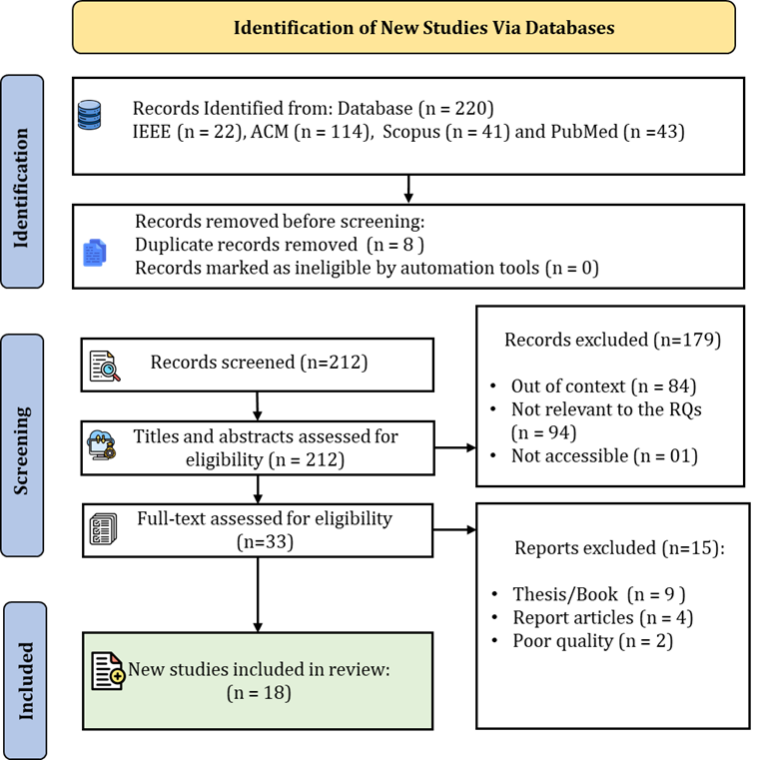
Preferred Reporting Items for Systematic Reviews and Meta-Analyses (PRISMA) flow diagram.

Our selection process, guided by PRISMA (Preferred Reporting Items for Systematic Reviews and Meta-Analyses) [24,25] guidelines as shown in Figure 2, allowed for a comprehensive understanding of the current state and future potential of BCI closed-loop systems in health care. We evaluated sources based on their methodology, innovation, significant findings, and overall relevance.

Our data analysis approach used a systematic data extraction method to rigorously analyze literature focused on BCI closed-loop systems integrated with ML and AI. This approach covered essential aspects such as the methods and parameters used in BCI systems, the ML algorithms used, challenges encountered, proposed solutions, and future research directions. Initially, we conducted an extensive literature review to identify pertinent studies. From this review, we developed a structured extraction matrix aimed at comprehensively capturing thematic elements critical to our study. The matrix included categories such as Title, Methods, Parameters, Machine Learning Algorithms, Challenges/Limitations, Proposed Solutions, Future Research Directions, and Title and Abstract Screening Score (0‐3). These scores would be averaged out among a panel of 3 researchers with a 2 being a “Yes” to our paper list. To validate our methodology, we conducted several validation steps. First, we pilot-tested the matrix with a small sample of 10 papers to ensure it effectively captured relevant information while excluding irrelevant details. Second, we aligned the matrix variable with our research questions to ensure clarity in data extraction. The finalized matrix, formatted in Microsoft Excel, allowed for a smooth, systematic, and comparative analysis across selected papers, including Full Text Screening Score (0‐3). This methodical approach enabled us to extract and synthesize data methodically, allowing anomalies and patterns to naturally emerge. Our synthesis and evaluation of articles were guided by their direct relevance to our study’s focus areas. This systematic approach ensured a robust analysis and provided a solid foundation for our literature review, as reflected in Table 1.

**Table 1.** Key algorithms and techniques commonly used in brain-computer interfaces.

Algorithm/technique	Role in BCIs^a^	Key applications	Advantages	References
Transfer learning (TL)	Feature extraction	Data alignment, spatial filtering	Improves robustness and accuracy	Shanechi [26]
SVM^b^	Classification	EEG^c^ signal classification	High performance in high-dimensional space	Gu et al [27]
LDA^d^	Classification	EEG signal classification	Computational simplicity, good performance	Gu et al [27]
ICA^e^	Preprocessing	Artifact removal	Isolates artifact components from neural signals	Tsai et al [28]
CNN^f^	Feature extraction	Emotion recognition, workload estimation	High accuracy in classifying brain activities	Mughal et al [29]
TSNN^g^	Feature extraction, classification	Neural activity classification	Effective in high-dimensional data	Shin et al [30]
RBM^h^	Dimensionality reduction	Mental state recognition	Learns underlying data structures	Wang et al [31]
Fuzzy models	Classification	EEG pattern classification	Handles uncertainty and imprecision	Wu et al [32]
GANs^i^	Data augmentation	Augmented data generation	Increases data robustness and accuracy	Tsai et al [28]

a BCI: brain-computer interface.

b SVM: support vector machine.

c EEG: electroencephalography.

d LDA: linear discriminant analysis.

e ICA: independent component analysis.

f CNN: convolutional neural network.

g TSNN: tree-structured neural network.

h RBM: restricted Boltzmann machine.

i GAN: generative adversarial network.

### Methods and Parameters Used in the BCI Closed Loop System (RQ1)

Many studies have explored BCIs with closed-loop systems, but a comprehensive survey focusing on the challenges associated with methods and parameters used in these systems is still lacking. This section addresses this gap by reviewing various preprocessing techniques and the parameters used in BCI closed-loop systems, highlighting their implications for neural activity monitoring and intervention.

#### Preprocessing Techniques

**Figure 3.**
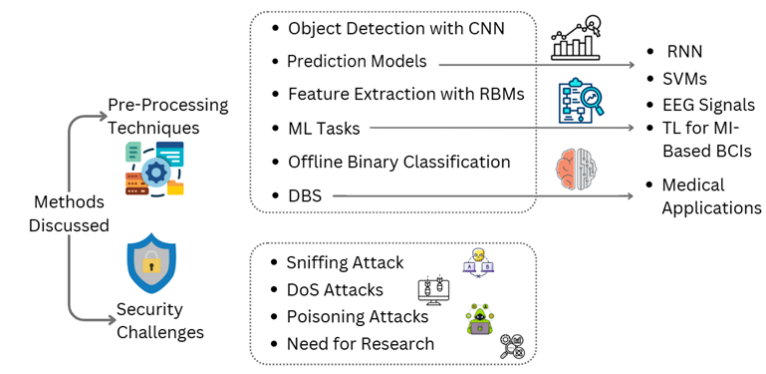
Different methods and parameters in the brain-computer interface closed-loop system. CNN: convolutional neural network; DBS: deep brain stimulation; DoS: denial of service; EEG: electroencephalography; MI: motor imagery; ML: machine learning; RNN: recurrent neural network; SVM: Support Vector Machine; TL: transfer learning;

The review identifies several effective methods and parameters that have demonstrated significant potential, as summarized in Figure 3. For instance, object detection is the paradigm for recognizing patterns using convolutional neural networks (CNN) [31], where it learns from more than a million images and can classify downstream objects in an image with high accuracy. This approach improves the ability to intermittently support real-time detection of nuanced neural activity and thus intervention. Likewise, Restricted Boltzmann Machines (RBMs) have been used to extract features for large-scale datasets [31]. Recurrent neural network (RNN) is a class of artificial neural network models that produce more accurate predictions than preferred direction and other systems like neuron-level readout methods including Poisson Process Velocity Tuning or generalized linear models (GLM). RNN can generate realistic simulations [33]. Support Vector Machines (SVMs) have been successfully used in small datasets, but their improvement to a larger accuracy level may be enhanced with Particle Swarm Optimization, particularly on the understanding of brain signals by means of EEG. BCI technology has been further refined by the categorization of different brain errors with SVMs. Motor Imagery (MI) is a mental process. MI starts from the thought of the movement of a body part. This activates different areas of the motor cortex and is commonly adopted for EEG-based BCIs. MI tasks performed by the users are sensed as EEG signals. TL makes use of source domain data to improve calibration in the target domain, which is a well-established technique used for improving MI-based BCIs [34]. In addition, offline binary classification is used to classify trials from target subjects. Currently, deep brain stimulation (DBS) is established as an effective treatment for conditions such as tremors, dystonia, and Parkinson disease. DBS also has shown promise in treating certain other types of chronic pain and psychiatric conditions, including neuropsychological tribulations. DBS is also being looked at as a possible pathway to the infusion of memory circuits and treatment avenues for dementia and Alzheimer disease.

#### Security Challenges

These technologies, hereafter referred to as potential neuromodulatory treatments for symptoms, have been demonstrated to be capable of driving neural signals. In fact, BCIs have a high risk of sniffing attacks, where an attacker can eavesdrop on network channels and preview unencrypted data. This vulnerability can be used to affect denial-of-service attacks, which, in the case of implanted BCIs, target battery depletion [35]. Poisoning attacks alter the behavior of a BCI machine learning system by providing it with malicious input. These inputs are generated to lead the respective outputs of a system into misleading neural signaling patterns. These types of attacks have catastrophic consequences, such as failing to trigger an alarm for a seizure. The resolution of these security challenges is paramount in ensuring the safe and efficient roll-out of BCIs. Further research and development are needed to improve the privacy/security properties of these systems so people with neurological conditions would be able to heavily rely on them.

### Effectiveness of the ML and AI Algorithms Used in the BCI Closed-Loop System (RQ2)

The effectiveness of ML and AI algorithms in BCI closed-loop systems is crucial for enhancing patient outcomes, particularly in applications related to neurorehabilitation and cognitive monitoring. These algorithms play a pivotal role in accurately interpreting neural signals, enabling real-time feedback and adaptive responses tailored to individual user needs. Their ability to analyze complex patterns in brain activity allows for improved signal classification and feature extraction, which are essential for ensuring reliable communication between the brain and external devices. Furthermore, the integration of effective ML and AI algorithms facilitates continuous learning and adaptation, ensuring that the BCI system evolves alongside the user’s cognitive state. This adaptability not only enhances the overall user experience but also promotes better engagement and efficacy in therapeutic interventions, making the technology a powerful tool in managing neurological disorders.

**Figure 4.**
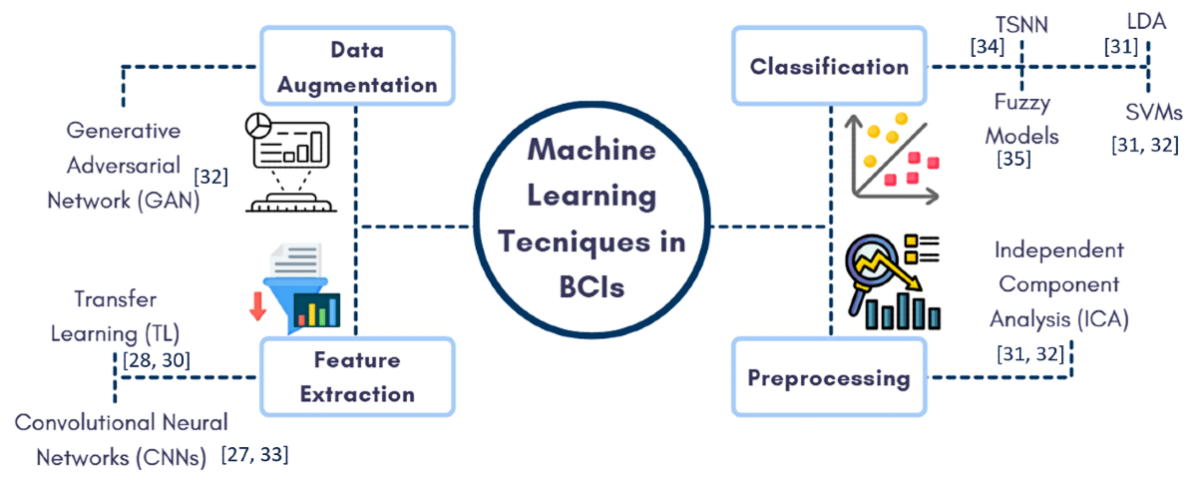
Machine learning techniques in brain-computer interface closed-loop systems [27,28,30-35]. BCI: brain-computer interface; LDA: Linear Discriminant Analysis; SVM: Support Vector Machine; TSNN: tree-structured neural network;

Figure 4 illustrates the key machine learning techniques used in BCI closed-loop systems. It categorizes these techniques into preprocessing (eg, Independent Component Analysis [ICA] for noise reduction), data augmentation (eg, generative adversarial networks [GANs] for expanding training data diversity), feature extraction (eg, CNN and transfer learning [TL] for identifying critical signal patterns), and classification (eg, SSVMs and linear discriminant analysis [LDA] for categorizing neural signals). These methods collectively improve the system’s effectiveness by refining the input data, enhancing model training with more varied data, extracting meaningful features, and accurately classifying neural patterns. This multistep approach enables closed-loop BCIs to achieve reliable real-time monitoring and intervention, making them more effective for health care and wellness applications.

In addition, Table 1 outlines the key algorithms and techniques commonly used in BCI systems, while Table 2 offers a detailed comparative evaluation of these machine learning approaches in closed-loop frameworks, emphasizing their applications, adaptability to neurological conditions, performance metrics, and computational complexity, supported by relevant literature.

**Table 2.** Comparative performance of machine learning techniques in brain-computer interface closed-loop systems.

Algorithm/ technique	Key use cases in BCI^a^ systems	Adaptability to neurological conditions	Avg accuracy / error Rate	Processing time / complexity	Reference(s)
SVM^b^	Motor imagery (MI), emotion recognition, EEG^c^ classification	Moderate adaptability; sensitive to intersubject variability	78%‐90% (low error in MI classification)	Fast on small datasets; efficient for real-time binary tasks	Gu et al, Tsai et al [27,28]
CNN^d^	Emotion detection, mental workload, EEG-fNIRS^e^ hybrid classification	High adaptability across subjects/sessions; handles complex patterns	>90% for workload/emotion tasks	High processing cost; ~300‐500 ms latency unless optimized	Mughal et al, Liang and Kao [29,33]
TL^f^	MI classification, cross-session calibration, cognitive decline monitoring	Highly adaptable; ideal for changing patient conditions (eg, AD^g^/ADRD^h^)	Reduces error up to 15% across domains	Moderate training cost; speeds up cross-subject adaptation	Shanechi, Belkacem et al [26,34]
LDA^i^	Basic EEG classification, passive BCI	Suitable for real-time low-power systems	~75%‐85% in EEG classification	Very low latency (<100 ms); lightweight	Gu et al [27]
ICA^k^	Noise reduction, preprocessing EEG/fNIRS	Improves SNR^j^, crucial for low-signal patients (eg, dementia)	Preprocessing only (not classifier)	Fast artifact removal; boosts downstream model accuracy	Gu et al, Tsai et al [27,28]
RBM^l^	Mental state recognition, feature learning	Good for poorly labeled, noisy data (common in AD/ADRD)	~80% in unsupervised tasks	Medium complexity; good for dimensionality reduction	Wang et al, Golshan et al [31,36]
Fuzzy models	EEG pattern classification, aBCIs	Handles uncertainty well; ideal for imprecise EEG from late-stage dementia	70%‐85% (context dependent)	Low to medium; interpretable rule-based outputs	Wu et al [32]
GAN^m^	Data augmentation for EEG/BCI model training	Improves performance in data-scarce or imbalanced domains	Indirectly improves downstream model accuracy	High training time; not used in real-time inference	Tsai et al [28]
TSNN^n^	Neurological disorder detection, adaptive BCI	Effective in high-dimensional, complex datasets	~88%‐92% in neural activity classification	Moderate-to-high, but hierarchical structure improves learning	Shin et al [30]
RNN^o^	Cognitive state prediction, BCI simulations	Well-suited for time-series EEG signal modeling	~85%‐90% (task dependent)	Computationally intensive; not ideal for all real-time apps	Liang and Kao [33]

a BCI: brain-computer interface.

b SVM: support vector machine.

c EEG: electroencephalography.

d CNN: convolutional neural network.

e fNIRS: functional near-infrared spectroscopy.

f TL: transfer learning.

g AD: Alzheimer disease

h ADRD: Alzheimer disease and related dementia

i LDA: linear discriminant analysis.

j SNR: signal-to-noise ratio.

k ICA: independent component analysis.

l RBM: restricted Boltzmann machine.

m GAN: generative adversarial network.

n TSNN: tree-structured neural network.

o RNN: recurrent neural network.

### Different ML Techniques in BCI Closed-Loop Systems

Machine learning algorithms have significantly enhanced the performance of BCIs. In that respect, one of the most influential techniques would be TL, which borrows knowledge from a source domain to perform better in a target domain. This is of particular importance to BCI, as the collection of data across sessions and subjects is often limited or variable [26]. TL has been successfully applied to data alignment, spatial filtering, feature selection, and classification tasks, dramatically improving the robustness and accuracy of BCIs across different conditions and subjects.

Complementing TL, SVMs have turned out to perform very well in high-dimensional spaces and have performed both linear and nonlinear classification using the kernel trick on data transformation [27]. SVMs have found wide applications in BCI applications, more specifically in classifying EEG signals. The SVM finds the optimal hyperplane that allows separation of different classes, thus always showing good performance in most BCI tasks, such as motor imagery classification and emotion recognition [28].

On the other side, LDA finds out the best separation between several classes by maximizing such a separation by choosing an appropriate linear combination of features. LDA has a nice balance between computational simplicity and performance for BCIs [27].

The role of ICA is paramount in preprocessing methods. ICA is one of the key tools that attempt to separate multivariate signals into additive, independent components [27]. Especially with BCIs, it is very good at isolating artifact components from the neural signals, hence improving the quality of data used for subsequent classification tasks. This step in preprocessing appreciably improves the accuracy of a number of BCI applications.

Moving to more complex models, CNNs have been very triumphant in visual and spatial data, including EEG and functional near-infrared spectroscopy (fNIRS) signals. CNNs are known for their emotional ability to build hybrid brain images that classify the activities taking place in the brain in a very accurate manner for the detection and interpretation of any complex neural pattern [29]. This is critical in applications like mental workload estimation and emotion recognition, where a spatial hierarchy in neural data may be critical for appropriate classification and analysis [33] .

Tree-structured neural networks (TSNN) combine decision trees and neural networks to provide the possibility of hierarchical feature extraction and classification. More importantly, these networks work quite effectively in relation to data: complex and high-dimensional. TSNNs are therefore able to yield promising results on the classification of neural activities and detecting symptoms of neurological disorders with a rich set of neural biomarkers [30]. This fills a gap in the field by providing an optimal balance between accuracy and computational efficiency, needed for real-time BCI applications.

RBMs have made some very great contributions to unsupervised scenes, where instances of the objective are to learn underlying structures of data [31]. In this case, RBMs learn with effective features and reduce dimensionality to improve the performance of classifiers on mental state recognition and motor imagery classification tasks [36].

On the other side, fuzzy models represent the uncertainty and imprecision of EEG data using fuzzy logic. Such models generate rules that are much closer to those resulting from human reasoning and hence are very suitable for processing nonlinear and nonstationary signals. Fuzzy models applied in BCIs include the so-called fuzzy inference systems (FIS) and fuzzy neural networks (FNNs) for classifying EEG patterns, offering both accuracy and interpretability [32].

Another extension to the toolkit of BCIs is GANs. It consists of two neural networks: a generator and a discriminator. These networks counteract in a framework, and each has an opposite goal in a zero-sum game setup. GANs’ application in BCIs is in augmented data generation for improving classifier training, more so when there is not enough data, as GANs increase the robustness, hence the accuracy, of BCI systems by bringing forth more training data. Improvements in these machine learning algorithms have increased the potential of BCIs not only on grounds of performance but also by opening new avenues for possible clinical and practical applications [28].

Table 1 outlines key methods used in BCIs, detailing their roles, applications, and benefits. Techniques like TL, SVM, LDA, and ICA enhance data preprocessing and classification, improving signal quality and performance. CNNs and TSNNs excel in feature extraction and classification of complex neural data, while RBMs and Fuzzy Models handle dimensionality reduction and uncertainty in EEG signals. GANs support data augmentation, boosting robustness and accuracy. These methods collectively optimize the processing of neural signals in closed-loop BCI systems.

TL, SVM, LDA, ICA, CNN, TSNN, RBMs, fuzzy models, and GAN techniques have helped in making BCIs effective and reliable. These algorithms help improve the capability of BCIs to better handle the user’s requirements, reduce calibration time, and realize more accurate and robust control of artificial limbs and other devices.

### Limitations in the Development and Implementation of the BCI Closed-Loop System (RQ3)

Some of the limitations facing BCIs’ development and implementation can be summarized as ranging from decoding algorithms through neural and behavioral measurements to computational constraints, as shown in Table 3. The table outlines various challenges associated with BCI technology and proposes corresponding solutions to address these issues. It covers aspects like improving neural signal decoding, enhancing sensor accuracy, and increasing the precision of behavioral measurements. These limitations show requirements for further research in terms of target setting and orientation of work for increasing effectiveness. Further explanations about these are as follows:

**Table 3.** Challenges and solutions in brain-computer interface development.

References	Associated problems	Proposed solutions
Bryan et al [37]	Neural signal decoding	Develop sophisticated algorithms
Jiang et al [38]	Accurate neural measurements	Advanced sensor technologies
Jiang et al [38]	Behavioral measurements	Improve granularity and precision
Gu et al [27]	Computational cost	Optimize algorithms and hardware
Gu et al [27]	Long calibration sessions	Develop efficient calibration methods
Merk et al [39]	Electrode design	Enhance ergonomic and reliable designs
Yue et al [35]	Decoding and encoding algorithm heterogeneity	Standardize methodologies
Wu et al [32]	Lack of long-term studies	Conduct long-term validation studies
Mughal et al [29]	Hardware limitations	Develop scalable hardware
Golshan et al [36]	Model generalization	Ensure models generalize to closed-loop conditions
Xavier et al [40]	Privacy and security	Implement robust security measures

#### Neural Signal Decoding

The challenging part of BCIs is the neural signal decoding into meaningful commands. This is due to the large array of neural signals requiring high accuracy; this becomes very challenging, especially in scenarios where they are either too noisy or highly variable across different cognitive states. This variability calls for sophisticated algorithms that can adapt to these changes and guarantee real-time performance [37]. Successful BCIs require accurate neural measurements; conventional methods generally have spatial and temporal resolution that is inadequate.

#### Behavioral Measurements

Behavioral measurements correlated with specific neural activities often experience imprecision and lack the fine detail necessary for comprehensive analysis. This limitation stems from the complexity of human behavior and the intricate relationship between neural processes and external actions. Standard measurement techniques may fail to capture the subtleties of these interactions, resulting in a loss of crucial information that could deepen our understanding of brain-behavior dynamics. Compounding this issue is the challenge posed by the time scale of behavioral dynamics; neural activities can change rapidly, often within milliseconds, while corresponding behavioral responses may take longer to manifest. This discrepancy makes it difficult to capture and analyze real-time correlations, as a sudden shift in brain activity may not immediately lead to observable changes in behavior, creating potential misalignments in data interpretation [38]. Consequently, the inability to accurately synchronize these fast-changing neural activities with their associated behaviors can hinder our understanding of cognitive processes and impair the effectiveness of interventions in areas like neurorehabilitation and BCIs. Addressing these challenges necessitates the development of advanced measurement techniques and analytical frameworks capable of capturing the nuances of both neural dynamics and behavioral responses.

#### Computational Cost

The high computational cost associated with processing and analyzing neural data presents a significant challenge in the development and implementation of brain-computer interface systems. TL techniques, when integrated with active BCIs, can incur substantial computational expenses due to the high-dimensional nature of neural data and the complexity of the models involved. This complexity poses a considerable burden on the real-time applicability of BCI systems, limiting their responsiveness and efficiency in practical scenarios. Furthermore, the current applications of TL in BCI research have primarily focused on binary MI classification problems, which restrict the versatility and scope of TL methods in broader contexts. As a result, the limitations of TL not only affect the computational feasibility of BCIs but also hinder their potential for more complex tasks, such as multi-class classification or real-time adaptive learning.

#### Long Calibration Sessions

One significant challenge associated with most MI-based BCIs is the extensive calibration sessions required before they can operate effectively. These lengthy calibration processes diminish the overall usability and practicality of BCIs, particularly in real-world applications where quick deployment is essential. To enhance the applicability of TL in everyday situations, it is crucial to develop more efficient calibration methods that can streamline the setup process and reduce the time commitment for users [27].

#### Electrode Design

An integral aspect of neural signal acquisition in BCIs is the design and fabrication of electrodes. Current electrode designs face significant challenges related to mechanical and electrical reliability, flexibility, and the speed at which they can accommodate various configurations. These issues can hinder the overall performance of BCIs, as unreliable electrodes may lead to inconsistent signal quality and compromised data accuracy. In addition, the pressure exerted by BCI headsets on the user’s head can result in discomfort during prolonged use, underscoring the need for improved ergonomic designs. Enhanced ergonomic considerations not only promote user comfort but also facilitate longer monitoring sessions, which are crucial for effective neural signal acquisition. By addressing these challenges in electrode design and headset ergonomics, researchers can significantly improve the functionality and user experience of BCIs, ultimately expanding their applications in clinical settings and enhancing the quality of life for individuals who rely on this technology [39].

#### Decoding and Encoding Algorithm Heterogeneity

The heterogeneity of decoding and encoding algorithms used in BCIs represents a significant challenge in the field. This diversity complicates comparisons across different closed-loop BCIs, as variations in purpose, methodology, and outcomes hinder the establishment of standardized benchmarks and best practices. Furthermore, the majority of existing studies tend to focus narrowly on cognitive neural features, often neglecting affective aspects of BCIs. This limited scope underscores the pressing need for larger, more comprehensive studies that encompass a broader range of neural activities and scenarios. By addressing the issues of algorithmic heterogeneity and expanding the research focus, the BCI community can enhance the comparability of findings, foster innovation, and ultimately improve the effectiveness and applicability of BCIs across various domains [35]. This will facilitate a deeper understanding of how different neural signals can be decoded and encoded, paving the way for more nuanced applications in both cognitive and affective realms.

#### Lack of Long-Term Studies

The absence of long-term studies significantly undermines the effectiveness of training BCI systems. Establishing a robust definition of a reinforcement signal is crucial, yet it raises ethical concerns, particularly when involving human participants. To mitigate these ethical dilemmas, it may be more appropriate to conduct initial experiments in nonhuman models, thereby sidestepping potential ethical issues. In addition, there is no assurance that human participants will interpret the feedback provided to them as a reward, complicating the training process further. This variability in interpretation can lead to inconsistent learning outcomes, making it challenging to develop reliable and effective BCI systems. Therefore, conducting comprehensive long-term studies is essential for refining training protocols, ensuring ethical compliance, and ultimately enhancing the overall effectiveness and applicability of BCIs in real-world scenarios [32].

#### Hardware Limitations

Hardware limitations pose significant challenges to the therapeutic effectiveness of BCIs, primarily through the need for higher channel counts and improved scalability. These requirements can result in the loss of critical information due to downsampling and channel selection processes, which may eliminate relevant neural signals necessary for accurate interpretation. In addition, there is often a considerable disparity between the sampling rate and the number of channels in EEG and fNIRS data, complicating the data analysis process. While proposed methodologies to address these issues aim to enhance data integrity, they frequently come with high computational costs and complexities that hinder their applicability in real-world settings. Consequently, overcoming these hardware limitations is crucial for advancing BCI technology, ensuring that it can deliver reliable and effective therapeutic outcomes for users [29].

#### Model Generalization

Another significant challenge in the development of BCIs is ensuring that models trained on open-loop data can effectively generalize to closed-loop conditions. The experiments necessary for this validation are often prohibitively expensive and time-consuming, which limits their widespread implementation. This highlights the critical need for real-time applicability of these models to facilitate the validation of adaptive deep brain stimulation (aDBS) systems [36]. Without the ability to efficiently transfer knowledge gained from open-loop scenarios to real-time closed-loop environments, the effectiveness and reliability of BCIs in practical applications remain in question. Thus, enhancing model generalization is essential for advancing BCI technology and ensuring its successful integration into therapeutic settings.

#### Privacy and Security

The issues surrounding privacy, security, and ethics are of paramount importance in the context of BCIs [41-44]. These systems are susceptible to various data breaches and cyberattacks, including cryptographic attacks, denial-of-service attacks, and sniffing attacks, which can compromise sensitive neural data and user information. Such vulnerabilities underscore the urgent need for robust privacy protection and comprehensive security measures to safeguard both the integrity of the data and the users’ personal information. In addition, ethical considerations surrounding the use of BCIs are critical, particularly regarding user privacy and informed consent. It is essential that users are fully aware of how their data will be used and are able to provide consent without coercion. Addressing these privacy, security, and ethical concerns is vital for the responsible development and deployment of BCI technologies, ensuring that they benefit users while minimizing potential risks and harms [40].

Ongoing collaborative research efforts are actively addressing the critical limitations identified in current BCI closed-loop systems. Among the most promising directions is the development of more sophisticated decoding algorithms capable of accommodating the inherent variability in neural signals across individuals and cognitive states. Improvements in neural and behavioral measurement precision—through advanced sensor technologies, multimodal signal integration, and robust signal processing methods—are also contributing to more accurate and responsive BCI systems. A key advancement involves the integration of TL with active BCIs beyond traditional binary classification, allowing systems to adapt across sessions and users while minimizing lengthy calibration times. In parallel, the design of more comfortable and reliable electrodes, alongside expanded studies into cognitive and affective dimensions of brain activity, is broadening the applicability of BCIs in both clinical and non-clinical environments. Furthermore, enhancing hardware scalability and addressing data loss due to downsampling remain essential for the therapeutic efficacy and widespread deployment of these systems. Ethical implementation, including user-informed consent and privacy-preserving frameworks, must be embedded into system design to ensure trust and adoption.

Recent advancements underscore how these challenges are being met through innovative and applied research. For instance, studies using TL and One-Shot Learning demonstrate that calibration requirements can be drastically reduced by reusing training data across users and sessions, enabling more efficient deployment in real-world environments [26,28]. In addressing cybersecurity concerns, researchers have proposed advanced encryption protocols and privacy-preserving neural computation strategies to mitigate sniffing, poisoning, and denial-of-service attacks—ensuring the confidentiality and integrity of neural data [35,40]. Notably, real-world applications such as Neuralink’s adaptive BCI and Tsai et al’s [28] secure closed-loop brain-machine interface exemplify successful responses to these challenges. These platforms leverage online tuning algorithms, secure data pipelines, and adaptive feedback systems to maintain robust performance while safeguarding patient data in both clinical and home care settings [35,45]. Together, these advancements highlight the growing maturity of BCI technologies and point toward a future in which user-friendly, secure, and scalable BCI systems are a practical reality.

### BCI Closed-Loop System-Based Framework for Longitudinal Monitoring of AD/ADRD Patients (RQ4)

AD/ADRD is a progressive neurological disorder, and one of the major causes of death among the older adults [46,47]. Therefore, it is important to acquire new solutions that can enhance the quality of life of patients and their caregivers as the number of people affected increases yearly. The use of BCI technology is considered one of the most promising approaches to this challenge, using state-of-the-art neuroimaging techniques and machine learning algorithms for continuous monitoring and diagnosis.

**Figure 5.**
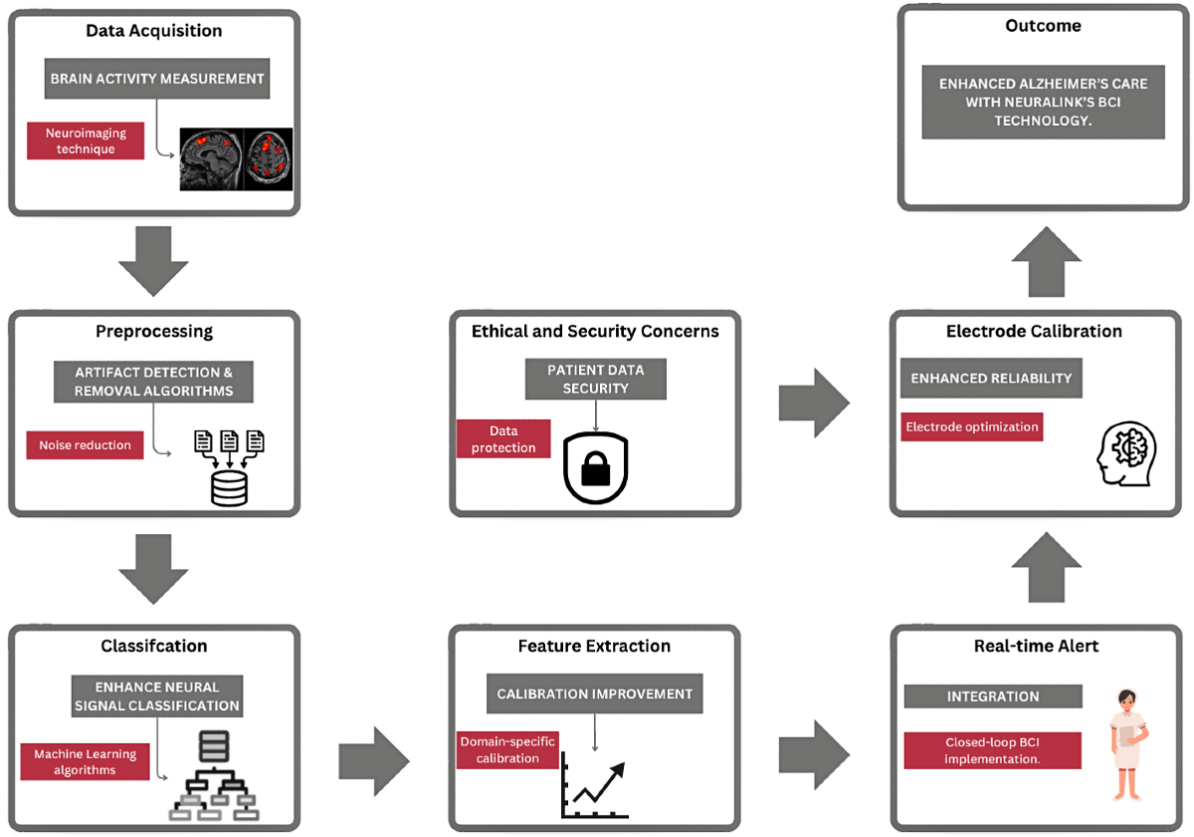
Closed-loop brain-computer interface framework for longitudinal monitoring of Alzheimer disease and related dementias patients.

The proposed framework shown in Figure 5 overcomes the complexity of decoding neural activity in AD/ADRD patients, who often lack the cognitive control normally required by conventional BCIs. It illustrates a closed-loop BCI framework for longitudinal monitoring of AD/ADRD patients, beginning with the acquisition of brain activity through neuroimaging techniques to capture critical neural signals. The collected data undergoes preprocessing, where noise is reduced using artifact detection and removal algorithms, ensuring high-quality signals. Ethical and security concerns are addressed by implementing robust data protection measures to safeguard sensitive patient information. Machine learning algorithms classify the neural signals, accurately distinguishing between various mental states, while feature extraction and domain-specific calibration improve the system’s precision. Electrode calibration ensures reliable signal acquisition, enhancing system performance. Real-time alerts integrate closed-loop monitoring for continuous observation, enabling timely interventions. Ultimately, this framework aims to improve Alzheimer patient care by leveraging Neuralink’s BCI technology [45] for better monitoring and intervention strategies. The different stages are further elaborated below:

#### Data Acquisition and Processing

The proposed framework is empowered for the correct interpretation of neural signals through sophisticated classifiers such as SVMs and LDA; it thus helps in the achievement of reliable monitoring and communication. Once brain activity data is collected through neuroimaging techniques like fMRI and EEG, preprocessing steps are crucial for artifact detection and noise reduction [48]. Advanced algorithms are used to filter out irrelevant data, ensuring that the subsequent analysis focuses on meaningful neural patterns.

#### Classification, Feature Extraction, and Electrode Calibration

In the classification phase, machine learning algorithms improve the deciphering of neural signals. In doing so, SVMs and LDA techniques can be used to classify the different mental states, giving insights into the patient responses and cognitive conditions. In this step, it is important to distinguish different neural activities and their behaviors. Feature extraction works on refining the accuracy and reliability of the classifications. These domain-specific calibration methods enable the tuning of the analysis so that the system learns the characteristics of the individual’s needs and variations in neural activity [48,49]. At this stage, electrode calibration is crucial for ensuring BCI system reliability. Optimized placement and configuration of electrodes ensures consistent data acquisition, reducing errors and thus enhancing the system’s overall performance.

#### Real-Time Alert

Real-time alerting of caregivers is another important factor in the framework. Data from both AD patients can be used to develop the alert system, ensuring that it can accurately identify deviations from normal neural activity. For instance, the classification accuracy reported in [48] indicates that the system can reliably distinguish between different mental states, which is crucial for triggering real-time alerts. Thus, this feature serves significantly in terms of preventing accidents or ensuring timely assistance from a medical point of view. This framework is aimed at enhancing the care of Alzheimer disease by integrating Neuralink’s BCI technology. The framework offers a strong and trustworthy tool for the betterment of the patient outcome and elongation of time that patients can spend with their loved ones by addressing the challenges of neural signal decoding, data security, and real-time monitoring.

### Ethical and Security Concern

After analyzing data, references indicate the most prominent symptoms of AD are severe deficits in communication, cognitive decline, and behavioral changes. Traditional BCIs require active participation; hence, they cannot be used with AD patients. For example, in [48] the authors highlight that traditional BCIs, requiring active control, are not suitable for AD patients due to their cognitive impairments. Instead, the study emphasizes the use of passive BCIs, which leverage preserved affective responses to facilitate basic communication and continuous monitoring [48]. This method makes use of detailed neuroimaging and machine learning components for cognitive and behavioral changes in order to provide such monitoring. Such continual assessment allows for early diagnosis and timely intervention that are crucial in managing AD progression [49]. In that respect, the BCI systems are vulnerable to data breaches and ethical misuses. The security aspects are core parts of the proposed BCI framework, evident in the position of ethical and security considerations at the very center of the framework diagram in Figure 5 [50]. This framework would hardwire strong measures for security against breaches and ethical misuse of information related to a patient. This involves encryption methods, secure storage, and tight access control that ensures only authorized personnel can view sensitive information. Despite technical possibilities, data protection and ethical guidelines are argued to exist, whereas BCI systems are weak in cryptographic, denial-of-service, and sniffing cyber-attacks [50]. Therefore, strong protection measures must be implemented. Implantable BCI devices give real-time data, thereby allowing caregivers to receive instant alerts on a patient’s condition. This approach will improve the quality of care and prevent emergencies.

The envisioned BCI framework addresses the most critical challenges of caring for the patient experiencing Alzheimer disease by efficiently combining advanced neuroimaging techniques with machine learning algorithms as shown in Table 4. In this regard, this approach is relevant for improving patient clinical outcomes while assisting caregivers in handling the complexities of Alzheimer disease management by enhancing neural signal classification, guaranteeing data security, and real-time monitoring [50].

**Table 4.** Mapping of framework components to challenges and supporting literature.

Framework component	Challenge addressed	Description	Supporting literature
Neural Signal Acquisition (EEG^a^/fMRI^b^)	Low signal-to-noise ratio (SNR); variability across sessions	Uses EEG and fMRI for high-resolution brain activity monitoring; requires preprocessing for noise	Gu et al, Liberati et al [27,48]
Preprocessing (eg, ICA)	Artifact contamination; real-time signal distortion	Independent Component Analysis (ICA) removes artifacts to improve signal clarity	Gu et al, Tsai et al [27,28]
Feature extraction and classification (SVM^c^, LDA)^d^	Inaccurate decoding of mental states	SVM and LDA used to classify neural patterns for real-time state detection	Shanechi, Gu et al [26,27]
Transfer learning (TL)	Lengthy calibration sessions; cross-user variability	Reduces setup time by leveraging previously trained models from similar domains	Shanechi, Belkacem et al [26,34]
Domain-specific calibration	Adaptability to individual neural profiles	Fine-tunes BCI^e^ parameters to individual characteristics	Shanechi, Liberati et al [26,48]
Real-time alert system	Lack of timely caregiver intervention	Monitors patient state continuously and sends alerts to caregivers during anomalies	Pisarchik et al, Liberati et al [45,49]
Passive BCI design	Limited cognitive engagement in AD^f^/ADRD^g^ patients	Enables nonintrusive monitoring based on implicit neural responses	Liberati et al, Liberati et al [48,49]
Security and ethical framework	Privacy risks; cyber threats; informed consent	Implements encryption, access control, and ethical safeguards for neural data	Yue et al, Xavier Fidêncio et al [35,40]
Scalable hardware integration	Usability and long-term deployment	Incorporates ergonomic, wearable sensors for home and clinical environments	Mughal et al, Merk et al [29,39]

a EEG: electroencephalography.

b fMRI: functional magnetic resonance imaging.

c SVM: support vector machine.

d LDA: linear discriminant analysis.

e BCI: brain-computer interface.

f AD: Alzheimer disease.

g ADRD: Alzheimer disease and related dementia.

## Discussion

### Principal Findings

This systematic review synthesized the current evidence from 18 studies on the integration of AI and ML within BCI closed-loop systems for neurorehabilitation, with a specific focus on AD/ADRD. The findings indicate that ML techniques such as TL, CNNs, and SVMs significantly enhance the performance of BCI systems by improving real-time signal classification, feature extraction, and cross-session adaptability. However, the translation of these technological advancements into widespread clinical practice is hampered by significant challenges, including signal variability, computational demands, lengthy calibration, and profound privacy concerns. The proposed framework for longitudinal AD/ADRD monitoring represents a promising, patient-centric application that leverages passive BCI paradigms to circumvent the cognitive demands of traditional systems.

### General Interpretation in the Context of Existing Evidence

Our findings on the efficacy of ML algorithms like CNNs and TL in BCI systems are strongly supported by the broader literature on AI in digital health. The high accuracy (>90%) of CNNs in classifying complex neural patterns for mental workload and emotion recognition aligns with their proven success in other pattern recognition domains, such as medical imaging. Similarly, the utility of TL in reducing calibration time and improving cross-subject generalization addresses a well-documented bottleneck in BCI research, echoing its successful application in other fields where data scarcity and individual variability are concerns. The review’s identification of passive BCIs as a solution for AD/ADRD patients is particularly insightful. This approach is consistent with a growing trend in digital biomarkers, which seeks to leverage implicit, continuous data from wearables and other sensors for early disease detection and monitoring, moving beyond active user participation. Furthermore, the emphasis on real-time, closed-loop feedback for neurorehabilitation is supported by neuroscientific principles of neuroplasticity. The ability of AI-enhanced BCIs to provide immediate, adaptive intervention is theorized to strengthen neural pathways more effectively than open-loop systems, a hypothesis that is gaining traction in stroke and spinal cord injury rehabilitation. Thus, the results of this review are not isolated but are part of a convergent evolution across AI, neuroscience, and clinical medicine toward more adaptive, data-driven therapeutic interventions.

### Limitations of the Included Evidence

While the reviewed studies demonstrate significant promise, the evidence base has several important limitations that temper the immediate readiness of these technologies for clinical deployment. The majority of included studies were small-scale, laboratory-based demonstrations. They often involved healthy participants or highly controlled patient groups, lacking the diversity and complexity of real-world clinical environments. This limits the generalizability of the reported high accuracy rates. In addition, as highlighted in the review, there is a pronounced heterogeneity in decoding algorithms, performance metrics, and experimental protocols across studies. The absence of standardized benchmarks makes it difficult to directly compare the performance of different ML models or BCI systems, hindering the identification of optimal approaches. Furthermore, there is a critical gap in long-term longitudinal studies. It remains largely unknown how these systems perform over months or years, how they adapt to disease progression, and whether improvements in signal classification accuracy translate into meaningful clinical outcomes, such as slowed cognitive decline or improved quality of life.

### Limitations of the Review Process

This review itself is subject to certain methodological limitations that should be acknowledged. Limiting the search to studies published between 2019 and 2024, while ensuring timeliness, may have excluded foundational or highly relevant older studies. Furthermore, while major databases were consulted, the exclusion of other potential sources may have led to the omission of pertinent research. Next, the review likely reflects a positive publication bias, as studies with null or negative results are less frequently published. This may create an over-optimistic picture of the current capabilities and reliability of AI-driven BCIs.

Despite following PRISMA guidelines and using a panel of researchers, the processes of screening titles/abstracts and extracting data into a matrix involve a degree of subjective judgment, which could have influenced the final selection and synthesis of the studies.

### Implications for Practice, Policy, and Future Research

The findings of this review have several critical implications across different domains:

For clinical practice: in the short term, AI-enhanced BCIs are most likely to find application as sophisticated diagnostic and monitoring tools in specialized neurology centers, aiding in the early and objective detection of cognitive impairment. The proposed framework for AD/ADRD provides a blueprint for developing caregiver alert systems, which could significantly reduce burden and improve patient safety in home-care settings. Clinicians should be aware of these emerging technologies to guide future patient care and manage expectations.

For policy and regulation: the security vulnerabilities and ethical dilemmas identified (eg, data privacy, informed consent for cognitively impaired users) demand urgent attention from policymakers and regulatory bodies like the FDA and EMA. New frameworks are needed to govern the security of neural data, which is arguably the most personal of all health information. Policies must be established to ensure equitable access and prevent misuse, defining clear guidelines for the ethical development and clinical validation of BCI technologies.

For future research: future work must transition from proof-of-concept to robust, clinically focused research. Key priorities should include rigorous, long-term trials with diverse AD/ADRD populations that are essential to validate efficacy and establish clinical utility. In addition, the BCI research community should collaborate to establish common data formats, reporting standards, and performance benchmarks to enable meaningful comparisons. Moreover, research must focus on developing more ergonomic, user-friendly, and low-power hardware that is suitable for prolonged use outside the lab. Creating interpretable ML models will be crucial for building trust among clinicians and patients, allowing them to understand the basis for the system’s classifications and decisions.

### Conclusion

This review systematically explored the role of BCI closed-loop systems in health care, with a specific focus on their potential to enhance neurological disorder detection and management through advanced ML and AI techniques. Addressing RQ1, we analyzed various methods and parameters used in BCI closed-loop systems, including signal acquisition, feature extraction, classification, and device output. Key preprocessing techniques such as ICA and TL were identified as crucial for reducing noise and improving signal quality. DBS was also highlighted as a promising intervention for neuropsychological disorders like AD and ADRD.

In evaluating RQ2, we examined the effectiveness of ML and AI algorithms in BCI systems. Techniques like Support SVM, CNN, and RNN demonstrated significant improvements in decoding neural activity, enabling more accurate classification of cognitive states. TL, in particular, showed promise in reducing calibration time, making BCI systems more adaptive to individual users. In addition, BCIs have expanded beyond disease detection, playing a pivotal role in cognitive enhancement, neurofeedback training, and assistive communication.

Despite these advancements, RQ3 highlighted several challenges in the development and implementation of BCI closed-loop systems. Key limitations include high computational costs, long calibration sessions, signal variability across individuals, and security risks such as Poisoning Attacks that could compromise neural signal integrity. Ethical concerns surrounding data privacy and the potential misuse of BCIs also remain pressing issues. Addressing these challenges requires advancements in real-time signal processing, improved sensor technology, and robust cybersecurity frameworks to protect patient data.

To answer RQ4, we proposed a BCI-based framework for longitudinal monitoring of AD/ADRD patients, integrating real-time neural signal acquisition, feature extraction, and ML-based classification for early cognitive decline detection. This framework incorporates real-time alert systems to assist caregivers in proactive intervention, enhancing patient outcomes. In addition, passive BCIs were identified as a viable alternative for patients with severe cognitive impairments, enabling continuous monitoring without requiring active user engagement.

To answer RQ4, we proposed a BCI-based framework for longitudinal monitoring of AD/ADRD patients, integrating real-time neural signal acquisition, feature extraction, and ML-based classification for early cognitive decline detection. This framework incorporates real-time alert systems to assist caregivers in proactive intervention, enhancing patient outcomes. In addition, passive BCIs were identified as a viable alternative for patients with severe cognitive impairments, enabling continuous monitoring without requiring active user engagement.

Building on these advancements, future research should prioritize the refinement of machine learning algorithms to better support real-time signal processing and adaptive learning in dynamic environments. Ethical considerations—such as user consent, data ownership, and secure data handling—must remain central to system design. Continued progress in these areas will be essential for creating scalable, secure, and user-friendly BCI systems that integrate seamlessly into daily life. Ultimately, these innovations will position AI-powered BCIs as transformative tools in improving care, independence, and quality of life for individuals with neurological disorders, particularly those living with AD/ADRD.

## Supplementary material

10.2196/72218 Checklist 1 PRISMA 2020 checklist.

## Abbreviations

ACM: Association for Computing Machinery
AD: Alzheimer disease
ADRD: Alzheimer disease and related dementias
AI: artificial intelligence
BCI: brain-computer interface
CNN: convolutional neural network
DBS: deep brain stimulation
EEG: electroencephalography
fNIRS: functional near-infrared spectroscopy
GAN: generative adversarial network
ICA: Independent Component Analysis
LDA: linear discriminant analysis
MI: motor imagery
ML: machine learning
PRISMA: Preferred Reporting Items for Systematic Reviews and Meta-Analyses
RBM: Restricted Boltzmann Machine
RNN: recurrent neural network
SVM: Support Vector Machine
TL: transfer learning
TSNN: tree-structured neural network
